# Student Medical Education Masterclasses: Diversifying the Academic Pipeline

**DOI:** 10.1111/tct.13850

**Published:** 2024-12-22

**Authors:** Victoria Collin, Megan Brown, Zaid Alsafi, Nicholas Sylvan, Ravi Parekh, Sonia Kumar

**Affiliations:** ^1^ Medical Education Innovation and Research Centre Imperial College London London UK; ^2^ School of Psychology University of East London London UK; ^3^ School of Medicine Newcastle University Newcastle UK; ^4^ Faculty of Medicine and Health University of Leeds Leeds UK

**Keywords:** academic career, careers, medical student, webinar, widening participation

## Abstract

**Background:**

There remains a lack of diversity among those pursuing clinical academic careers. Structural inequalities, discrimination and a paucity of relatable role models can disadvantage minoritised students, hindering their educational experiences and career opportunities. Innovative and effective approaches are needed at an undergraduate level to address this problem, ensuring the pipeline is representative, diverse and inclusive.

**Approach:**

To help address this challenge, in 2021, we developed a free online ‘medical education masterclass series’ for 250 UK medical students, with students from minoritised backgrounds particularly encouraged to apply. The masterclasses explored topical themes in medical education through seminars and workshops featuring diverse guest speakers.

**Evaluation:**

To evaluate the programme, students were invited to submit an anonymised reflection and complete a semi‐structured interview exploring how their perceptions of medical education may have changed following attendance of the masterclasses. A total of 18% responded, and data were thematically analysed.

**Results:**

Students reflected on how the sessions broadened their understanding of medical education beyond their own curriculum experiences. Students described acquiring skills and building networks to further develop their interest in medical education. The desire to make medical education more inclusive was identified as a key priority for students, and students felt empowered to advocate for positive change within their own institutions.

**Implications:**

Providing students with opportunities to explore medical education through such masterclasses has the potential to raise awareness and address some of the barriers experienced by minoritised students. We would advocate for similar initiatives to be developed in other clinical academic areas to increase diversity.

## 1 Background

There are known inequalities relating to gender, ethnicity, age and disability in clinical academic careers [1]. Progression within clinical academic careers is often described as ‘leaky’, as academics are lost from the profession as they try to advance [1]. This is particularly problematic for women and Black doctors who are underrepresented, particularly in senior positions [2, 3, 4]. Discrimination, inflexible working patterns, lack of clarity in the career path, scarcity of relatable role models/mentors and differential awarding due to biases in assessments are attributed to these disparities [1, 4, 5].

Addressing these issues supports the creation of an academic workforce that reflects the diversity of its student and patient populations, which is critical for enhancing belonging [4, 6] and addressing health inequalities [7]. Making a career in medical education accessible to *all* medical students is therefore essential. Multiple barriers have been identified that may hinder medical student engagement in medical education, including opportunities to undertake research or teaching or relatable medical school role models [8]. Furthermore, there is often a lack of an established career path in many universities, which can make medical education seem a less attractive option [9].

Multiple barriers have been identified that may hinder medical student engagement in medical education, including opportunities to undertake research or teaching or relatable medical school role models [8].

There is often a lack of an established career path in many universities, which can make medical education seem a less attractive option [9].

Medical students from lower socioeconomic backgrounds also report lacking the social capital and awareness of the need to network and capitalise on opportunities to advance their careers [10]. Exposure to academic opportunities, peer mentoring and access to role models can be an effective means to help students access a career in medical education [11]. Initiatives to encourage medical students to consider academic careers include opportunities to undertake teaching and research, for example, during electives, intercalated degrees, special study modules or through specific workshops and conferences [12]. However, these have typically involved a small number of students engaged in studentships [13].

To help address some of these barriers, we developed a free, online ‘medical education masterclasses’ for up to 250 UK medical students, with students from minoritised backgrounds [14] particularly encouraged to apply. We sought to evaluate how attendees' perceptions of medical education may have changed following the masterclasses.

## 2 Approach

The masterclasses comprised of seven half‐day online sessions spread over 2 weeks in July/August 2021, which aimed to inform and engage students in medical education (Box 1).

Box 1 **Aims and rationale for medical education masterclass series.**

The masterclasses aimed to:

1. Provide an opportunity for students to gain a deeper understanding of academic medical education and explore key topical areas within the literature.
2. Empower students to further explore academic medical education in their own contexts. This may range from progressing their own professional development, to advocating for change in their own institutions, to considering a future career within academic medical education.

The masterclasses were designed to be as inclusive as possible, by being free to attend, online, and delivered in the summer to minimise educational disruption. Students from minoritised* backgrounds were particularly encouraged to apply by promoting the masterclasses through specific networks, societies and student groups.

*We're using the following definition of minoritised as put forward by Selvarajah et al. [14] ‘individuals and populations, including numerical majorities, whose collective cultural, economic, political and social power has been eroded through the targeting of identity in active processes that sustain structures of hegemony’.

These sessions featured a diverse range of guest speakers, interactive workshops and panel discussions on core themes in medical education (Table 1).

**TABLE 1:** Agenda/timetable.

Day 1	Starting your journey in medical education Introduction from team and interactive small group sessions on exploring key issues in medical education, careers in medical education and how to level the playing field.
Day 2	Digital health and education^a^ Overview and facilitated group discussions on topics such as wearable technology, telemedicine, digital exclusion and the future of digital health.
Day 3	Diversity and inclusion^a^ Facilitated discussions, including a Q&A with an expert panel on diversity and inclusion and how it relates to medical education and healthcare
Day 4	Preparation for practice: learning through longitudinal experiences^a^ Presentation introducing longitudinal integrated clerkships (LICs), followed by discussions about merits and challenges of LICs and a Q&A panel with expert speakers.
Day 5	Professional identity^a^ Presentations and small group discussions on experiences of professional identity formation at medical school.
Day 6	Coaching^a^ Overview of coaching and how it is applicable to medical education, followed by a workshop on practising coaching techniques in small groups
Day 7	Final session Recap and reflections from the masterclass, opportunity to submit an anonymous reflection.

^a^ These themes were chosen as they align to the hosts' (Medical Education Innovation and Research Centre) key areas of interest. Throughout the masterclasses, students were asked to reflect on how medical education may be improved to address issues surrounding inequalities, workforce retention, community priorities and social accountability. This was to encourage students to develop a broader understanding of what medical education entails.

## 3 Evaluation

After the masterclasses, students were invited to submit an anonymised, structured, written reflection in response to three questions (Box 2).

Box 2. 2

1. How have your perceptions of medical education and research changed following attendance at the masterclass? Has this affected your interest in a career in medical education?
2. How might you apply what you had learnt from the masterclasses in your own medical school?
3. What are the key priorities that you think medical education should focus on?

Students were also invited to take part in a semi‐structured interview to explore their answers. The reflections and interviews were transcribed verbatim and thematically analysed using a reflexive approach [15]. A total of 237 students attended the masterclasses, from 23 medical schools, representing over half of UK medical schools (Table 2).

**TABLE 2:** Participant demographics.

Demographic characteristic	%	*n*
Gender^a^		
Female	69.9	114
Male	27.0	44
Non‐binary	1.8	3
Prefer not to say	1.2	2
Ethnicity^a^		
Asian/British Asian	48.1	158
Black/African/Caribbean/British	9.7	16
Multiple/mixed ethnic groups	9.1	15
White	23.1	38
Any other ethnic group	7.4	6
Prefer not to say	2.4	4
Year of study		
1st year	19.8	47
2nd year	20.7	49
3rd year	22.4	53
4th year	21.9	52
5th year	12.2	29
6th year	2.9	7

^a^ From those who completed the voluntary EDI form, *n* = 163.

A total of 42 students submitted anonymous reflections, and we interviewed five of these students. Through pooled analysis of the reflections and interviews, we identified four key themes:

1 Broadening understanding of medical education

Many students possessed a narrow view of medical education prior to the masterclasses, focused on teaching methods/skills delivery and were unaware of wider aspects such as professional identity formation, addressing diversity and inclusion issues, and social accountability.

I began to see medical education in a different light and saw it from angles I had never realised before. (Reflection 13)

Students acknowledged the broad impact that medical education has for the workforce and students:

I thought medical education was simply ‘educating doctors’. Now, I realise it is so much more … … their future work in healthcare, how they treat their patients, how they are perceived ….and how they can change medicine for the better. (Reflection 39)

Students attributed their limited understanding of the breadth and depth of medical education to a lack of exposure during medical school, contributing to the perceived elusiveness of medical education career choices:

I feel there's still a little bit of mystery kind of surrounding it, people might know that it exists but … … they do not know how to get involved or how to even have a career in it. (Interview 1).

Furthermore, the critical role that medical education has in teaching medical students about the wider aspects of patient care was acknowledged. Students felt they should be more than a ‘repository of knowledge’ (Interview 2) and instead develop skills within curricula that allow doctors to connect with their patients and develop empathy.

2 Motivation for pursuing a career in medical education

For some, increasing their knowledge and developing new networks were motivations for signing up to the masterclasses.

I had been interested in medical education, but… I did not quite know what it entailed or how I'd be able to work it into my career … the thought of gaining more knowledge and getting some connections was enough to motivate me to go. (Interview 1)

Students' interest was cultivated through attendance at the masterclasses, fuelled by topics that students could personally identify with:

areas … that really sparked my interest were digital health, professional identity and diversity & inclusion. These topics especially resonated with me and have influenced me to try and make a positive change via a career in medical education. (Reflection 12)

3 Personal development

The value of networking and connecting with others with an interest in medical education was noted as beneficial by many students. For some, this was seen as a way to mitigate against their lack of social capital:

I do not have that traditional medicine background to sort of bolster me ….it feels like everyone else has got an advantage and I do not…the biggest highlight is meeting other like‐minded students ….It helps to form a lot of really good connections … So hopefully that'll just open more doors …. (Interview 1)

Students also spoke about applying some of the knowledge and skills they acquired during the masterclasses in their everyday studies, for example, using coaching principles to facilitate their learning or enhance patient interactions.

4 Empowering students to advocate for change

During the masterclass series, students recognised the power of their voices to drive change in their institutions:

I have also realised that we as students hold a great amount of power to lead and promote positive changes to our education. Prior to this I did not think that I could make much of a difference. (Reflection 33)

For some students, this shift in perception manifested as a sense of empowerment, with students reporting how they could use their own lived experience to promote grassroots change in medical education:

… … I've always thought about it, but encouraged me to actually take action…I'm [now] part of a widening participation scheme … … Because coming from a disadvantaged background myself I found it difficult. (Interview 5)

## 4 Implications

Previous research has identified a lack of equity in accessing and pursuing a career in medical education [1]. By attending a series of free, accessible online masterclasses, medical students gained knowledge, skills and confidence, which they believed would further empower them to pursue medical education opportunities during and beyond their undergraduate studies. Through exploring topics in medical education, students increased their knowledge and challenged their perceptions of what medical education entails. By exposing students to medical education in their undergraduate studies and highlighting its broad applicability, their interest was ignited, considering medical education as a viable career option. This echoes previous research showing that interest in medical education is fostered by early exposure and opportunities to learn more about and demystify medical education careers [8].

By exposing students to medical education in their undergraduate studies and highlighting its broad applicability, their interest was ignited, considering medical education as a viable career option.

Crucially, the masterclasses also allowed students to connect with others in the field and build contacts. This was particularly salient for those from minoritised backgrounds in medicine, who often lack the social capital afforded to students from more privileged backgrounds [10]. Ensuring that medicine is more inclusive was a priority for many students, including widening accessibility, diversifying the academic workforce and curriculum, and reducing health inequalities for patients. This is consistent with the literature advocating for *increasing* diversity in medical healthcare professionals from a social justice perspective [7].

Crucially, the masterclasses also allowed students to connect with others in the field and build contacts.

The masterclasses also allowed students to come to the realisation that they had a voice or power that they could use to address some of the challenges that they themselves or others may have faced at medical school. This was particularly evident from students who identified as not being a ‘typical’ medical student who saw engaging with medical education as an opportunity to support medical students like themselves and challenge the status quo. Students identified different avenues to achieve this, including advocating for their peers, offering mentoring, engaging with faculty–student evaluations, applying for new roles or using their existing platforms in student societies or other networks.

The masterclasses also allowed students to come to the realisation that they had a voice or power that they could use to address some of the challenges that they themselves or others may have faced at medical school.

### 4.1 Limitations

To minimise educational disruption and increase student numbers, the masterclasses were scheduled over the summer months. However, medical students who work over the summer may not have been able to apply. As the reflections were voluntary, we may have captured the views from a particularly motivated sample.

### 4.2 Conclusion

By engaging in the masterclasses, students felt empowered to pursue further opportunities in medical education and advocate for change. We would suggest that similar initiatives be developed in other fields of clinical academia where challenges in striving for equitable participation also exist. Recommendations for such initiatives are provided in Box 3.

Box 3 **Recommendations for future masterclasses.**

Recommendation 1: Designing the masterclasses to be accessible:

- Timetabling outside of term‐time
- Free/low cost attendance
- Consider online/hybrid delivery
- Advertise through channels to reach a diverse student population (e.g., widening participation channels and social media)

Recommendation 2: Ensure there is diversity among the speakers:

- Allow for a range of different perspectives to be heard
- Fosters representation and allows for concordant role‐modelling

Recommendation 3: Create opportunities for networking and collaboration:

- Embed interactive small‐group activities in the programme
- Strive to keep groups consistent to foster relationship building.

Recommendation 4: Create a community to share future opportunities for student involvement and networking.

- As part of the masterclasses, we offered students the opportunity to take part in a medical education research competition, with the winning two inter‐institutional submissions being supported to complete the project with supervision and funding.

## Author Contributions

**Victoria Collin:** conceptualization, investigation, writing–original draft, methodology, formal analysis, writing–review and editing. **Megan Brown:** investigation, writing–original draft, methodology, formal analysis, writing–review and editing. **Zaid Alsafi:** writing–original draft, formal analysis, writing–review and editing. **Nicholas Sylvan:** writing–original draft, writing–review and editing, formal analysis. **Ravi Parekh:** conceptualization, writing–review and editing, supervision. **Sonia Kumar:** supervision, writing–review and editing, conceptualization.

## Ethics Statement

The Education Ethics Review Process Team at Imperial College London granted ethical approval (EERP2021‐098).

This article presents independent research commissioned by the National Institute for Health Research (NIHR) under the Applied Health Research (ARC) programme for North West London. The views expressed in this publication are those of the author(s) and not necessarily those of the NHS, the NIHR or the Department of Health and Social Care.

## Conflicts of Interest

The authors declare no conflicts of interest.

## Data Availability

Due to ethical constraints, we are not able to share our data.
